# Fibrinolytic Activity and Dose-Dependent Effect of Incubating Human Blood Clots in Caffeic Acid Phenethyl Ester: *In Vitro* Assays

**DOI:** 10.1155/2015/627471

**Published:** 2015-01-15

**Authors:** Abuzar Elnager, Rosline Hassan, Zamzuri Idris, Zulkifli Mustafa, Nadiah Wan-Arfah, S. A. Sulaiman, Siew Hua Gan, Wan Zaidah Abdullah

**Affiliations:** ^1^Department of Haematology, School of Medical Sciences, Universiti Sains Malaysia, Health Campus, 16150 Kubang Kerian, Kelantan, Malaysia; ^2^Department of Neurosciences, School of Medical Sciences, Universiti Sains Malaysia, Health Campus, 16150 Kubang Kerian, Kelantan, Malaysia; ^3^Unit of Biostatistics and Research Methodology, School of Medical Sciences, Universiti Sains Malaysia, Health Campus, 16150 Kubang Kerian, Kelantan, Malaysia; ^4^Department of Pharmacology, School of Medical Sciences, Universiti Sains Malaysia, Health Campus, 16150 Kubang Kerian, Kelantan, Malaysia; ^5^Human Genome Centre, School of Medical Sciences, Universiti Sains Malaysia, Health Campus, 16150 Kubang Kerian, Kelantan, Malaysia

## Abstract

*Background*. Caffeic acid phenethyl ester (CAPE) has been reported to possess time-dependent fibrinolytic activity by *in vitro* assay. This study is aimed at investigating fibrinolytic dose-dependent activity of CAPE using *in vitro* assays. *Methods*. Standardized human whole blood (WB) clots were incubated in either blank controls or different concentrations of CAPE (3.75, 7.50, 15.00, 22.50, and 30.00 mM). After 3 hours, D-dimer (DD) levels and WB clot weights were measured for each concentration. Thromboelastography (TEG) parameters were recorded following CAPE incubation, and fibrin morphology was examined under a confocal microscope. *Results*. Overall, mean DD (*μ*g/mL) levels were significantly different across samples incubated with different CAPE concentrations, and the median pre- and postincubation WB clot weights (grams) were significantly decreased for each CAPE concentration. Fibrin removal was observed microscopically and indicated dose-dependent effects. Based on the TEG test, the Ly30 fibrinolytic parameter was significantly different between samples incubated with two different CAPE concentrations (15.0 and 22.50 mM). The 50% effective dose (ED50) of CAPE (based on DD) was 1.99 mg/mL. *Conclusions*. This study suggests that CAPE possesses fibrinolytic activity following *in vitro* incubation and that it has dose-dependent activities. Therefore, further investigation into CAPE as a potential alternative thrombolytic agent should be conducted.

## 1. Introduction

For decades, fibrinolytic (thrombolytic) therapy has been an important means of establishing reperfusion [1]. Thrombolytic agents, which activate plasminogen to form plasmin, lead to accelerated lysis of thrombi. Thrombolytics are applied in the treatment of acute myocardial infarction, acute pulmonary embolism, and deep vein thrombosis. For example, thrombolytic therapy administered within 12 hrs of a heart attack may increase the chances of survival. However, limitations to the use of thrombolytic therapy include perceived or definite contradictions and intracranial bleeding in cardiovascular diseases [2–5]. Therefore, thrombolytic therapy is only limited to very few pathological conditions that have limited complications.

Thrombolytic drugs used clinically are basically divided into three generations. First-generation thrombolytics include streptokinase (SK) and urokinase (UK). Second-generation drugs include tissue plasminogen activator (t-PA) and single-chain urokinase-type plasminogen activator (scu-PA, or prourokinase, pro-UK). The third generation includes novel agents produced from the first- or second-generation thrombolytic agents based on modern molecular biology techniques. However, even with widespread use, they have been reported to potentially cause bleeding complications, have a short half-life, exhibit low fibrin specificity, are costly, may contribute to allergic reactions, and require administration of large therapeutic doses [2–6].

Propolis is a resinous hive product collected by honeybees from various plant sources. Depending on its source and age, it may vary from yellow-green to dark brown. It contains many compounds including amino acids, phenolic compounds, and flavonoid compounds [7–15]. Caffeic acid phenethyl ester (CAPE) is a plant-derived phenolic compound that is an active component of honey and propolis from honeybee hives [16]. CAPE has antithrombotic (by modulating tissue factor expression at a posttranscriptional level in endothelial cells) [17], antiviral, antibacterial, anti-inflammatory [18, 19], anticancer [20], antiatherosclerotic, antioxidative [16, 19, 21], immunostimulatory, and tumor growth inhibition activities. CAPE has also been shown to be a potential inhibitor of some enzymes including ornithine carboxylase, 5-areductase, protease, lipoxygenase, cyclooxygenase, and human immunodeficiency virus- (HIV-) 1 integrase [22].

Previous studies [5, 7, 23] have reported various fibrinolytic enzymes that are contained in different Asian foods including Tofuyo, Korean Chung kook-Jang soy sauce, Japanese Natto, edible honey mushrooms, and cheonggukjang kinase 3–5 (CGK). These enzymes have been shown to convert proenzyme plasminogen into serine protease plasmin and play a vital role in fibrinolysis [5, 7, 23]. In addition, the* Bacillus subtilis* K42 isolated from soybean flour has also been reported to have thrombolytic potential [24]. Some additional examples include the use of a recombinant CGK-rich fraction isolated from cheonggukjang [25] as a thrombolytic and fibrinolytic agent and* B. Subtilis* LD-8547 culture broth, which is another useful thrombolytic agent [26].

Currently, there are limited studies on the* in vitro* assessment of fibrinolytic potentials of various compounds. Some evidence of fibrinolysis has been found in whole blood (WB) clots that exhibited different patterns of fibrin morphology when viewed on a confocal microscope using streptokinase [27]. Plasma clotting has also been described as an alternative method to study* in vitro* fibrinolytic activity. One study utilized plasma clots and washed red blood cells (RBCs) at different concentrations to determine the effects of RBCs on fibrin clot structure by confocal microscopy. The authors noted that different concentrations led to heterogeneity in fibrin network structure [28, 29]. Another study reported that CAPE has time-dependent* in vitro* fibrinolytic activity [30]. However, to date, no study has been conducted to investigate the dose-dependent effects of CAPE on fibrinolysis. If the dose-dependent effect of CAPE can be determined, it will provide evidence for further investigation into the use of CAPE as a potential new fibrinolytic agent.

## 2. Materials and Methods

The present study was approved by the local institutional ethical committee, Universiti Sains Malaysia, with the ethical numbers: FWA 00007718 and IRB Reg number: 00004494. The fibrinolytic activity of CAPE at different concentrations was measured using a previously reported method, which was used to demonstrate that CAPE has time-dependent fibrinolytic activity at 3, 6, and 9 hrs [30]. The WB clot lysis method to study fibrinolysis was performed according to previously reported procedures [27, 30]. Various* in vitro* fibrinolytic assessments were performed including D-dimer tests (DD), WB clot weight measurements, thromboelastography (TEG) assays, and morphological assessment of fibrin by confocal microscopy.

TEG was performed on WB clots because it allows a global assessment of hemostatic activities. TEG overcomes the limitations of conventional coagulation tests and provides an inclusive overview of hemostasis, starting from early thrombin production to the formation of fibrin strands and fibrinolysis. Moreover, TEG with defined parameters reflects the integrity of particular hemostatic components [31–34]. DD is a specific product of fibrinolysis, and the quantitative measurement of DD assesses the extent of fibrinolysis at the molecular level. Moreover, modern automation of the coagulometer permits the direct quantitation of DD. WB clot weight is an indirect marker of fibrinolysis that measures clot disintegration, which reduces the clot weight over time. Lastly, morphological assessment of fibrin is best done by confocal or electron microscopy, which could reveal the microchanges in fibrin structure during the fibrinolytic process.

Standardized human WB clots were incubated for 3 hrs in pooled platelet-poor plasma (PPP) (as a normal control), 5% dimethyl sulphoxide (DMSO) (as a solvent control), or CAPE at different concentrations (3.75, 7.50, 15.00, 22.50, or 30.00 mM). DD was measured from the supernatant of PPP-, DMSO-, and CAPE-treated clots (at the various stated concentrations). WB clots were weighed before and after incubation, and WB clots were examined for fibrin structure using a specific dye under a confocal microscope (Carl Zeiss, Jena, Germany). In this study, different CAPE concentrations from 3.75 to 30.00 mM were investigated based on previous reports in which 15 mM CAPE exhibited fibrinolytic activity [30]. The WB clots and PPP were prepared first, followed by fibrinolytic assessment of CAPE by the WB clot lysis method.

*(i) WB Clot Preparation*. Standardized WB clots were prepared from 4.50 mL of venous blood derived from a healthy volunteer with blood group O positive (*n* = 10). The whole blood was then transferred into three different preweighed, sterile siliconized, plain glass tubes (12 × 75 mm). Each tube contained 1.50 mL WB without anticoagulant and the blood was allowed to clot at room temperature for 10 min. Prior to the incubation in water bath, the blood containing tubes were covered with parafilm to avoid contamination and haemolysis by water evaporation. After that, the tubes were incubated in a temperature-controlled water bath (Grant SUB6, England) at 37°C for 3 hrs to ensure complete clot retraction and separation of the clot from the edges of the tubes. After clot retraction the serum was carefully removed from the edges of the glass tube using a Pasteur pipette without disturbing the clot. Finally, the tubes were completely dried using filter paper (125 mm). The weight of the tube together with the clot was determined using an electronic balance (AND FR-200 MK II, Japan). The WB clot weight was estimated by calculating the difference between the weight of the tube containing the clot and the weight of an empty tube. The tubes with WB clots were properly labeled for further experiments. The WB clot weight used in this study was standardized to 0.70 ± 0.15 grams.

*(ii) Preparation of Normal Pooled Plasma (for Normal Control and as Diluents)*. Human pooled PPP was prepared and was strictly processed by collecting 9.00 mL of WB into two trisodium citrate tubes (4.5 mL each) from participants with O blood group (*n* = 20). Blood was drawn using an evacuated tube system with multisample green sterile needle (21 G). Immediately after blood collection, the samples were centrifuged (Eppendorf, 5810 R, Germany) at 1500 ×g for 15 min at room temperature. Then, the supernatant was centrifuged at 1200 ×g for 15 min. The procedure was carried out according to the Clinical Laboratory Standardization Institute (CLSI) guidelines for coagulation tests.

The PPP from all the participants was pooled and sample integrity was tested using coagulation parameters. The tests included prothrombin time (PT), activated partial prothrombin time (APTT), fibrinogen, DD level, and factor VIII activity using STA-R Evolution coagulation analyzer (Stago, Asnières, France). Von Willebrand factor antigen was also measured using specific reagents of the system based on the protocol provided with kits for the ACL Elite PRO (Belford, USA). The tests were performed to ensure that normal PPP was obtained for the study. The yielded pooled PPP was then aliquoted into small volumes in different centrifuge tubes (BD biosciences, San Jose, USA) and was stored at −80°C deep freezer for 6 months according to the CLSI guideline for coagulation assays.

### 2.1. WB Clot Lysis Method for Fibrinolytic Assessment of CAPE

Commercially available pure CAPE powder was purchased from Sigma Company (Aldrich, USA). The CAPE solution was prepared in DMSO solution as per the previously reported procedure and manufacturer's instructions [30]. In the present study, a 3.6 M CAPE stock solution was prepared and stored at −20°C. The stock solution was thawed at room temperature and used for the preparation of dilutions to measure fibrinolytic activity.

Different concentrations of CAPE (3.75, 7.50, 15.00, 22.50, and 30.00 mM) were prepared using pooled PPP as the diluent. The WB clots were immersed in each dilution and incubated in a temperature-controlled water bath at 37°C for 3 hrs. After incubation, the pooled PPP was transferred into a microcentrifuge tube (bullet tube) using a Pasteur pipette after gentle shaking of the clot. The plasma was centrifuged at 1200 ×g for 5 min, and the supernatant was separated for DD measurement using coagulation analyzer as mentioned above. After removal of the plasma, the clots were weighted by calculating from the differences of the weight of the tube before incubation and the weight after incubation (WB clot weight = weight of tube containing WB clot before incubation − weight of tube containing WB clot after incubation). A similar procedure was repeated for 10 times to measure the changes of DD and WB clot weight following treatment with CAPE. The 50% effective dose (ED50) was calculated using Excel (Microsoft, USA). Similar tests were also performed for the controls as described below.

### 2.2. WB Clot Incubated in Pooled PPP (Normal Control) and 0.5% DMSO (Solvent Control)

One milliliter of each control (pooled PPP and 0.5% DMSO diluted in pooled PPP) was added to a tube containing a WB clot (method described as above), which was then covered by parafilm. The tubes were incubated in a temperature-controlled water bath at 37°C for 3 hrs. Following incubation, the plasma was transferred to a microcentrifuge tube (bullet tube) using a Pasteur pipette. The weight of each glass tube containing the clot after incubation was measured and the clot weight was calculated by taking the difference between the preincubation WB clot weight and the postincubation WB clot weight. The plasma collected in the bullet tube was centrifuged at 1200 ×g for 5 min, and the supernatant was separated for DD quantitation using coagulation analyzer (as described below). The whole procedure was carried out 10 times to measure clot weights and DD levels.

### 2.3. Quantitation of D-Dimer

Quantitation of DD levels was performed using the STA-R Evolution coagulation analyzer and was photometrically determined by the immunoturbidimetric method using the Liatest kit (Stago, Asnières, France). This test was performed according to the manufacturer's protocol.

### 2.4. Confocal Microscopy Protocol and Staining Procedure

Confocal microscopic observations of WB clots were conducted as per a previously described method [27] with some slight modifications [28, 29, 35] to suit the conditions in this study. Briefly, stock solution was prepared by dissolving 5 mg of fibrinogen in 3.30 mL of 0.1 M sodium bicarbonate (NaHCO_3_) (pH 8.3) at room temperature. The solution was stored at 4°C for further use. Subsequently, a working solution was prepared by adding 100 *μ*L of fibrinogen (stock) dye to distilled water (6 mL) followed by solubilization with occasional gentle mixing for one hour using a mechanical stirrer. Fibrinogen fluorescence dye [Alexa Fluor 488 human fibrinogen conjugates (F-13191)] purchased from Molecular Probes (USA) was used in this study.

The working solution (0.3 M) of merocyanine 540 fluorescent (erythrocyte dye) was prepared by dissolving 3.4 mg in 20 mL of distilled water. Phosphate-buffered saline (PBS) was used as a washing buffer in this study. Another buffer of 0.1 M NaHCO_3_ was used as the diluent for the fibrinogen fluorescence dye.

For confocal microscopic observations of WB clots, venous blood (5 mL) obtained from O blood group volunteers was used. WB (180 *μ*L) was placed in each well of an 8-chamber polystyrene vessel with a tissue culture-treated glass slide (BD falcon cultures, USA) according to the ratio used for WB clot lysis described above.

The chamber was allowed to stand for 10 min at room temperature and was then incubated in a temperature-controlled water bath at 37°C for 3 hrs to ensure complete clot retraction. After incubation, the serum was completely removed from the chambers. Then, retracted WB clots without CAPE (group 1, controls) or in 120 *μ*L of CAPE (group 2) at different concentrations (3.75, 7.50, 15.00, 22.50, and 30.00 mM) were added to the WB clot in the chambers. The chamber was labeled accordingly and incubated in a temperature-controlled water bath at 37°C for 3 hrs. The CAPE (group 2) was carefully removed, and the WB clots were washed with PBS for 3–5 min. The washing was then repeated 3 times. The fibrin fibers were labeled with 600 *μ*L of freshly prepared working solution of the primary antibody Alexa Fluor 488 human fibrinogen conjugate F-13191 before incubation at room temperature and were then left in the dark for 15 min to avoid exposure to direct light.

After 15 min, the stain was removed, and the clot was washed 3 times with PBS. Then, it was incubated with buffer at room temperature for 5 min. Afterwards, the buffer was removed, and 600 *μ*L of the merocyanine label (RBC-emission wavelength 520 nm; Molecular Probe USA) was added to the WB clot for red blood cell (RBC) staining. It was then incubated at room temperature for 45 min in the dark. The dye was washed using PBS for 3–5 min, and the procedure was repeated 3 times. Antifade fluorescence mounting medium was added, followed by examination on a confocal microscope. Images of fibrin and RBCs for each group were obtained on a Pascal 5 Axiovert inverted laser confocal microscope with a 63x lens using an argon and helium-neon (HeNe) laser. For confocal imaging of retracted WB clots, Alexa 488 labels fibrin fibers green, and the merocyanine 540 fluorescent dye (MC-540) labels the RBC membranes are red [27].

### 2.5. Thromboelastography (TEG) Assay Procedure

A computerized coagulation analyzer (TEG model 5000; Haemoscope, Niles, IL 60714, USA) was used to test the citrated blood positioned in oscillating cups at 37°C with the pin hanging from the torsion wire into the blood sample. The fibrin strand formation joins the motion of the cup to the pin. The trace is then displayed on the computer screen where the deviation in the trace is proportional to the clot strength. This method was based on the methods described in previous studies and reports [31, 32, 36]. Citrated whole blood (4.5 mL) from nine healthy volunteers (*n* = 9) was tested, and the specimens were carefully checked for fibrin clots prior to testing to detect other contaminating materials. One milliliter of blood was pipetted into each kaolin tube for the normal control or was incubated with CAPE at different concentrations (15.00 and 22.50 mM) or 0.5% DMSO as a solvent control. For the TEG study, only two concentrations were assessed. These tubes were incubated for 3 hrs at 37°C. Subsequently, 20 *μ*L of calcium chloride (0.2 mol/L) was added to a standard specimen cup. After the sample was adequately mixed, 340 *μ*L of citrated whole blood was added to the cup, and the assay was run for at least 90 min until completion of the measurement of clot lysis at 30 min.

TEG parameters that represent different aspects of hemostasis were measured (*R*, *K*, *α*, MA, and LY30). *R* represents the time which elapsed from baseline until the first fibrin clot was formed. The cut-off point is when the amplitude reaches 2 mm. Clot kinetics (*K*) are measured from the time of clot formation until an amplitude of 20 mm is reached. Alpha (*α*) is the angle starting from the point of the clot reaction that represents the acceleration of fibrin production and cross-linking. MA is the maximum amplitude of the graph from the clot kinetic activity, which reflects the greatest strength of the clot. It is a function of both the fibrin and the platelet dynamic properties. LY30 represents the clot lysis 30 min after MA is reached, indicating the rate of fibrinolytic activity [31, 34].

### 2.6. Statistical Analysis

Statistical analyses were performed using PASW Statistics 20 (SPSS, Chicago, IL). Fibrinolytic data using DD were analyzed using a parametric one-way analysis of variance (ANOVA) test because the data were normally distributed. The WB clot weights before and after plasma incubation and following CAPE treatment were compared using a nonparametric Wilcoxon Signed Rank test (because the data were not normally distributed). For the TEG parameters, the normality test also revealed that the results obtained for the parameters were not normally distributed; therefore, the data were analyzed using a Kruskal-Wallis test. Multiple Mann-Whitney tests with Bonferroni correction were applied. The level of significance was set as <0.05.

## 3. Results

A preliminary study was conducted using various incubation times to investigate the fibrinolytic effect of CAPE on DD levels, and the results of DD tests performed after 1 to 9 hrs of incubation were compared. The incubation times at 3 hrs and above showed consistent results and significant differences in DD (data not shown here). Previously, CAPE was shown to have time-dependent fibrinolytic activity [30]. In the current study, the fibrinolytic effects exhibited by CAPE at different concentrations were investigated and showed significant differences in mean DD after incubation with various CAPE concentrations: 3.75, 7.50, 15.00, 22.50, and 30.00 mM (except at 15.00 versus 30.00 mM and 22.50 versus 30.00 mM).

**Figure 1:**
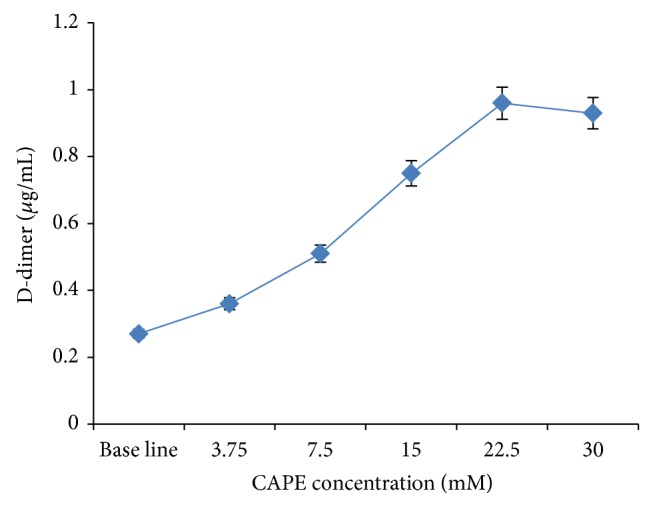
Fibrinolytic activity of CAPE.

There was no significant difference in mean DD levels between the normal control (PPP) 0.27 (SD = 0.03) and 0.5% DMSO solvent control 0.27 (SD = 0.05). On the other hand, there was a reduction in DD after incubation of 22.50 mM CAPE (Figure 1), with a 50% effective dose (ED50) of CAPE of 1.99 mg/mL.

There was a statistically significant reduction in postincubation clot weight compared to preincubation clot weight using the different CAPE concentrations. Furthermore, the weights of the WB clots following CAPE incubation were significantly lower than the preincubation clot weights at all concentrations. The median WB clot weights between the various CAPE concentrations showed no significant differences except for 22.50 compared with 3.75 mM, 7.50 mM, and 15.00 mM.

### 3.1. Confocal Microscopy

**Figure 2:**
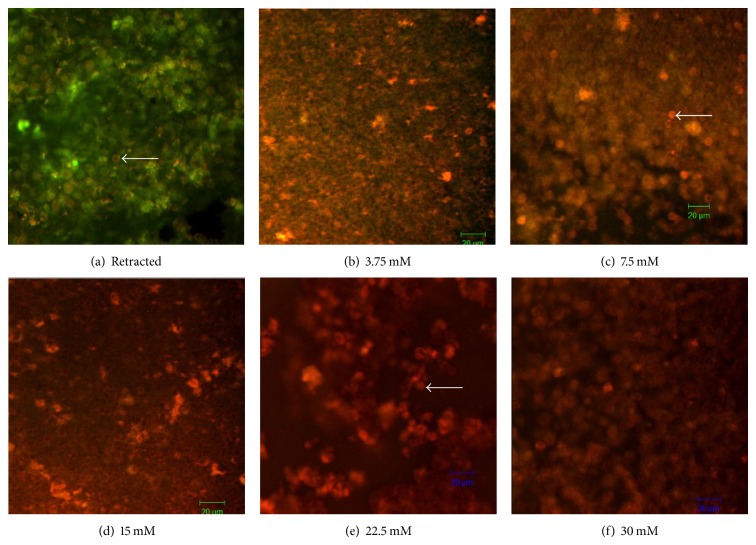
Confocal images for retracted WB clots treated with CAPE. (a) Image of normal retracted WB without CAPE treatment. The figure demonstrates fibrin binding the RBCs in the retracted WB clot (control); the white arrow is pointing to the RBCs. (b) 3.75 mM CAPE; the fibrin is reduced on the RBCs. (c) 7.50 mM CAPE; the fibrin level is less than in 3.75 mM CAPE sample. (d, e, and f) 15.00, 22.50, and 30.00 mM CAPE, respectively; the fibrin is completely absent, leaving behind only RBCs that appear red in color (63x, magnification bar = 20 *μ*m).

The* in vitro* experiments were performed using retracted WB clots incubated in pooled PPP and CAPE at different concentrations. The fibrin fibers were removed from the WB clots in a manner proportionate to the concentrations of CAPE. At the lower concentrations of 3.75 and 7.50 mM, there was less fibrin than in the untreated clots (control). At these two concentrations, the RBCs appeared orange in color due to the combination of red (RBCs) and green (fibrin) coloring which completely disappeared at the higher concentrations, 15.00, 22.50, and 30.00 mM, where only red coloring was present, reflecting the presence of RBCs only and the absence of fibrin (Figure 2).

### 3.2. Thromboelastograph (TEG) Findings

TEG parameters are presented in Table 1.

 
*R* time: sample incubated with 22.50 mM CAPE exhibited a significantly different *R* time than the normal controls but not with 15.00 mM CAPE.
 
*K* time: there were no significant differences between the normal controls, 15.00 mM CAPE samples, or 22.50 mM CAPE samples.
 Alpha angle (*α*): there were no significant differences between the normal controls, 15.00 mM CAPE samples, or 22.50 mM CAPE samples.
 Maximum amplitude (MA): there was a significant decrease in MA for samples incubated with either 15.00 or 22.50 mM CAPE compared with that of the normal controls.
 The LY30: the LY30 trended upward from the normal control to the 15.00 mM CAPE samples, but statistically insignificant. There were significant differences between the normal controls and the 22.50 mM samples as well as the 15.00 and 22.50 mM samples.

All TEG parameters in the normal and 0.5% DMSO controls showed insignificant differences.

## 4. Discussion

The present study used previously reported* in vitro* WB clot lysis methods of fibrinolytic activity assessment to investigate the concentration-dependent fibrinolytic activity of CAPE* in vitro* [27]. TEG parameters, including a fibrinolytic parameter (LY 30), were also measured to assess the effects of CAPE. This study was an extension of a previous* in vitro* study that reported that CAPE has time-dependent fibrinolytic activity [30]. Plasma and solvent controls (DMSO) were used to control the effect of fibrinolytic activity that occurs spontaneously with time and to exclude solvent-induced fibrinolysis [30, 37]. The DD level tended to increase with increasing CAPE concentration. In the early stages of this study, it was found that three hours of CAPE led to significant and consistent changes in fibrinolytic activities (e.g., DD levels) than did one or two hours of CAPE incubation. However, incubation with less than 3.75 mM CAPE had no significant effect on fibrinolysis, although effects below the sensitivity threshold for the methods used to assess fibrinolysis* in vitro* cannot be discounted. Blood tested* in vitro* with a DD concentration greater than 0.50 *μ*g/mL strongly indicates fibrinolytic activation in the blood clot because the threshold DD level for normal pooled plasma is 0.50 *μ*g/mL (based on the local lab reference value).

The study demonstrated that after incubation, different concentrations of CAPE led to fibrinolytic activity in the human blood clot model. A statistically significant difference in the mean DD levels was observed between samples incubated with different CAPE concentrations with the exception of 15 mM compared with 30 mM and 22.50 mM compared with 30.00 mM. These exceptions may be attributed to either a limiting factor imposed by the fibrinolytic process or the fact that these concentrations gave a rather similar dose effect. Incubation with 22.50 mM CAPE led to higher DD levels (peak), which declined progressively with increased CAPE concentration. This decline was most likely due to the peaking in the enzyme activity (plasminogen) which gradually subsided. Mechanistically, fibrinolysis usually involves the binding of plasminogen to fibrin and conversion to plasmin (an active form of the plasminogen) by tissue-type plasminogen activator (tPA). Plasmin then cleaves the fibrin at specific sites producing fibrin degradation fragments that can be used as markers of fibrinolysis by measuring DD [38]. In the present study, the observed increase in DD is most likely due to plasminogen activation by CAPE. However, the exact mechanism of activation needs to be determined in future studies especially* in vivo*. The sigmoid curve in Figure 1 reaches a plateau after 22.5 mM which actually explains the rate-limiting factor in the fibrinolytic procedure.

Our study is the first to report on the ED_50_ of CAPE, which was determined to be 1.99 mg/mL. Previous reports of the ED_50_ of various compounds, drugs, or thrombolytic agents that have been studied for effects on plasma clot lysis have utilized clot turbidity as a marker to determine clot mass following incubation with 6% bovine serum albumin- (BSA-) phosphate-buffered saline (PBS) and pooled normal human plasma. The ED_50_ of fresh clot lysis for BSA and pooled normal human plasma were found to be relatively low at 128 and 180 IU/mL (urokinase), 0.3 and 0.2 *μ*g/mL (t-PA), 215 and 1371 IU/mL (streptokinase), 60 and 91 U/mL (plasminogen-streptokinase activator complex), 664 and 996 U/mL (reteplase), and 0.2 and 0.2 *μ*g/mL (tenecteplase), respectively [39]. However, the methods used to assess clot lysis were different; the Microtiter Plate Clot Lysis Assay was used [39].

The relationship between DD level and CAPE concentration was nonlinear above 22.50 mM CAPE, most likely because of the rate-limiting effects of the fibrinolytic enzyme. This finding correlated with a previous study that reported a nonlinear increase in DD concentration using plasma clots in recombinant tissue plasminogen activator- (rt-PA-) based fibrinolysis [40]. In this study, retracted WB clots were used as they are more suitable than plasma clots for evaluating coagulation parameters, as previously reported [27, 30, 41]. The WB clots also mimic* in vivo* clot formation better than plasma clots, as RBCs and white blood cells (WBCs) are part of the clot structure.

The antioxidant, immunomodulatory, anti-inflammatory, antiplatelet, and time-dependent fibrinolytic activities of CAPE have previously been reported [30, 37, 42–44]. Our study is the first to investigate the fibrinolytic activity of CAPE at different concentrations using* in vitro* methods. This study is similar to a previous study that used tPA-induced fibrinolysis at a higher temperature to cleave the fibrinogen and fibrin in a thrombus into fragments D and E, as quantified by measurement of the DD concentration. DD is a specific marker for fibrin degradation [40]. This study agreed with the earlier reports that also used DD, hemoglobin, and radioactive ^125^I-fibrinogen to evaluate clot lysis activity. These different parameters tended to increase [30, 45, 46], indicating evidence of fibrinolytic activity.

In the present study, the WB clot weight was used to investigate clot lysis activity. The median WB clot weight post-CAPE incubation was significantly lower than the median pre-CAPE incubation clot weight for every CAPE concentration tested. These results agree with previously published studies [3, 27, 30, 40, 47, 48]. However, no significant differences in weight were observed among samples incubated with different CAPE concentrations, although significant weight reduction was observed in some of the groups. The sensitivity of using WB clot weight to detect fibrinolytic activity has been shown to be lower than the sensitivity achieved using DD levels [27]. Samples incubated with 22.50 mM CAPE were significantly different than samples incubated with 3.75, 7.50, or 15.00 mM CAPE, which may indicate that 22.50 mM CAPE has a more obvious effect on clot lysis* in vitro* that is similar to the effect of 30.00 mM CAPE.

The current* in vitro* study assumed a concentration-dependent CAPE effect, in accordance with the former study that demonstrated a dose-dependent CAPE effect (at 1, 5, 10, and 20 mg/kg) when injected intravenously in rats (*in vivo*) as determined by evaluating heart rate and blood pressure. The lethal dose of CAPE was found to be 20 mg/kg for rats [7]. Previous studies have reported that fibrinolytic enzymes can be found in a variety of foods, such as Japanese Natto, Tofuyo, Korean Chungkook-Jang soy sauce, and soybean flour (*Bacillus subtilis* K42). Another strain of* Bacillus subtilis* LD-8 47 was isolated from douchi, a traditional Chinese soybean-fermented food, and XZNUM 00004 was extracted from* Streptomyces* sp. and edible honey mushrooms [4, 5, 23, 24, 26]. Furthermore, a recombinant CGK study in mice demonstrated protection from death due to pulmonary embolism using various doses of this substance including 130, 260, and 520 mg/kg [25].

Microscopy is best suited to determine fine details and information of fibrin clot structures [27, 28]. In the present study, WB clots incubated in different concentrations of CAPE showed fibrin removal from RBCs after 3 hrs of incubation that disappeared at the higher concentrations. A previous study revealed increased porosity of fibrin when treated with thrombin and factor Xa inhibitors using 3D confocal microscopy [49]. This study agreed with a former study that performed qualitative assessment of WB clot morphology using confocal microscopy [27]. The effect of CAPE on fibrin imaging was evaluated by comparing treated samples with untreated fibrin as shown by the representative image in Figure 2. The WB clot confocal images demonstrated that the fibrin fibers were prominent and surrounded by RBCs in the control sample but not in the CAPE-treated clots. The fibrin network was suddenly lost in WB clots and no fibrin fibers were visible when clots were incubated with different CAPE concentrations. At concentrations of 3.75 and 7.50 mM, a reduced fibrin meshwork was observed as an orange color (indicating a combination of green fibrin and red RBCs). Ultimately, the fibrin fibers vanished or disappeared with higher concentrations of CAPE (15.00, 22.50, and 30.00 mM).

A fibrin clot is a three-dimensional network made up of fibers with unique mechanical properties that explain the correlation between clot structure and the mechanical and fibrinolytic stability of clots [38]. In this study, the fibrin fibers were removed faster in CAPE-incubated clots than in plasma-treated (control) clots, as shown in a previous study [27] using streptokinase. This observation reflects the effect of penetration of CAPE into the WB clot. The structure of the fibrin networks underwent significant changes in architecture during the lytic process [28, 29, 49].

In this study, only two concentrations of CAPE (15.00 and 22.50 mM) were used for the TEG assay. These CAPE concentrations were selected because they led to significant changes in DD levels (Figure 1). The TEG parameters for clots incubated with 15.00 and 22.50 mM CAPE were different, indicating different hemostatic activities. There was no solvent effect from the DMSO in which the CAPE was prepared.

The TEG parameter *R* time was lower in both CAPE-incubated samples than in the normal controls. Based on the pattern of the TEG tracing, CAPE may activate coagulation factors* in vitro* shortening the *R* time. Another TEG parameter, *K* time, showed insignificant differences between the normal controls and different CAPE concentrations. A possible reason for this observation is that CAPE does not possess anticoagulant properties and, therefore, does not lead to differences. Another TEG parameter, alpha angle (*α*), tended to increase at 22.50 mM CAPE. Previous reports have described TEG changes under selected conditions [34, 36]. CAPE has been reported to have antiplatelet properties. In this study, however, no antiplatelet effect of CAPE was shown based on the alpha angle parameter, although there was an increase in alpha angle values when a higher dose of CAPE (22.5 mM) was used.

The next TEG parameter, MA, was significantly reduced in samples incubated with both concentrations of CAPE compared with normal controls. MA values, however, showed continuous reduction over time, which may indicate the fibrinolytic effects of CAPE [34] because MA is a function of both fibrin and platelet dynamic properties. Therefore, the antiplatelet and fibrinolytic effect of CAPE may have contributed to the reduced MA value. Lastly, the TEG parameter LY30 showed the fibrinolytic activity of CAPE using TEG method. Additionally, fibrinolysis can be indicated by a normal *R* time and a disproportionately decreasing MA value over time. Fibrinolytic activity measure by the TEG parameter LY30 might not correlate perfectly with DD levels as fibrin degradation produces different split products other than DD [36].

In this study, different techniques have been used to assess the fibrinolytic activity of CAPE at different concentrations. Although all the results showed evidence of fibrinolytic effect of CAPE, there are still some slight differences. For example, DD detects fibrinolytic activity at the molecular level in the presence of split products of fibrin degradation, while clot weight is a crude procedure which may not be sensitive enough to indicate CAPE's fibrinolytic effects. TEG parameter is a good tool which can show fibrinolysis activity based on the dynamic changes of the blood clot. The morphological assessment detects fibrin dissolution very early even at the lowest dose of CAPE. The confocal microscopy which detects structural changes of fibrin may be influenced by many factors including CAPE concentration, buffers, dyes, and WB clot size used for confocal microscopy.

In this study, the dose response curve (Figure 1) showed a range of CAPE concentrations. Although practically this may not be possible (very high concentration was included, i.e., 30.00 mM of CAPE), natural product containing CAPE may illustrate synergistic effects with other compounds, in particular with other phenolic substances. Therefore, variabilities in fibrinolytic effects may be yielded, which is hoped to be overcome when high CAPE concentrations are used. Although not reported here, the fibrinolytic effects of crude propolis using DD has been shown to be higher than that of CAPE alone. Therefore, further investigations are required in the future to confirm this finding.

There are many expected limitations in* in vitro* studies. In this study, the effects of pH and ionic strength in the tests system were not critically evaluated. However, normal and solvent controls were included to mitigate this effect. The normal control and solvent control were included. The effects of the pure compound which is free from influences of other factors should be investigated and reconfirmed.

## 5. Conclusion

This study demonstrates that at 3 hours of incubation CAPE has dose-dependent fibrinolytic activity* in vitro.* CAPE has a fibrinolytic potential to gradually lyse WB clots* in vitro*, suggesting that it could be used as an alternative thrombolytic agent in the future. More studies are required to evaluate the biological effects of CAPE and to confirm its fibrinolytic potential for* in vivo* use.

## Figures and Tables

**Table 1:** Median interquartile range (IQR) for TEG findings in the normal controls and CAPE.

Variable	Normal control median (IQR)	CAPE 15.00 mM median (IQR)	CAPE 22.50 mM median (IQR)
*R*-time (min)	5.90 (1.45)	3.50 (2.70)	3.05 (1.90)
*K*-time (min)	1.90 (0.40)	2.30 (2.10)	1.55 (1.03)
*α* angle (degree)	64.80 (6.00)	61.50 (17.20)	69.60 (12.85)
MA (mm)	60.60 (3.65)	41.60 (19.80)	49.70 (10.15)
LY30 (%)	0.40 (1.15)	1.70 (5.05)	21.35 (11.85)

*R*: reaction time; *K*: clotting time; *α*: angle; MA: maximum amplitude; LY30: clot lysis.
